# Genome-Wide Identification of *BES1* Gene Family in Six Cucurbitaceae Species and Its Expression Analysis in *Cucurbita moschata*

**DOI:** 10.3390/ijms24032287

**Published:** 2023-01-24

**Authors:** Minyan Xu, Yanping Wang, Mengting Zhang, Meng Chen, Ying Ni, Xuewei Xu, Shengkai Xu, Yuting Li, Xin Zhang

**Affiliations:** National Engineering Laboratory of Crop Stress Resistance Breeding, School of Life Sciences, Anhui Agricultural University, Hefei 230036, China

**Keywords:** genome-wide analysis, *BES1*, brassinosteroids, transcription factor, Cucurbitaceae, *Cucurbita moschata*

## Abstract

The *BES1* (BRI1-EMSSUPPRESSOR1) gene family play a vital role in the BR (brassinosteroid) signaling pathway, which is involved in the growth and development, biotic, abiotic, and hormone stress response in many plants. However, there are few reports of *BES1* in *Cucurbita moschata*. In this study, 50 *BES1* genes were identified in six Cucurbitaceae species by genome-wide analysis, which could be classified into 3 groups according to their gene structural features and motif compositions, and 13 *CmoBES1* genes in *Cucurbita moschata* were mapped on 10 chromosomes. Quantitative real-time PCR analysis showed that the *CmoBES1* genes displayed differential expression under different abiotic stress and hormone treatments. Subcellular localization showed that the most of CmoBES1 proteins localized in nucleus and cytoplasm, and transactivation assay indicated 9 CmoBES1 proteins played roles as transcription factors. Our analysis of BES1s diversity, localization, and expression in Curcubitaceae contributes to the better understanding of the essential roles of these transcription factors in plants.

## 1. Introduction

The growth and development of plants are inseparable from various protein network systems, and transcription factors are one of these proteins. Transcription factors can control chromatin and transcription by recognizing specific nucleotide sequences to form complex systems that guide the gene expression and play unique roles in plant developmental stages [1]. *BES1* (BRI1-EMS-SUPPRESSOR1) is a plant-specific transcription factor identified via a mutant bes1-D; this mutant completely inhibits the *bri1 dwarf* phenotype and exhibits a constitutive BR (brassinosteroid, steroidal phytohormone) response [2]. The BES1 protein family has conserved protein structures: a putative nuclear localization sequence, a highly conserved amino-terminal domain, a phosphorylation site for GSK-like kinase BIN2 (brassinosteroid insensitive 2), and a carboxyl-terminal domain [3]. Phosphorylation and dephosphorylation of the BES1 protein mediate BR signal transduction [4,5].

BRs are plant-specific polyhydroxylated steroid hormones, which are classified as primary growth-promoting hormones, regulating multiple processes of plant growth including seed germination, cell elongation and division, photomorphogenesis, vascular differentiation, stomatal formation, leaf vein formation, reproductive development, and cell senescence [6,7,8,9,10,11]. BRs can protect plants from various biotic and abiotic stress, including insect and pathogen attacks, low and high temperatures, drought, and salinity stresses [12,13,14,15]. When BR is absent in cells, BES1 is phosphorylated by BIN2 and remains in the cytoplasm without entering the nucleus, so its DNA-binding activity is inhibited [16,17,18,19,20,21]. When BR is at a high level, it is sensed by the cell surface receptor kinase BRI1 (brassinosteroid insensitive 1), and then transmits the signals to the *BES1/BZR1* transcription factor [22]. BES1 is dephosphorylated by PP2A, accumulated from the cytoplasm into the nucleus, and bound to the promoter’s E-BOX (CANNTG) to induce gene expression [23]. *BZR1* is a transcriptional repressor that binds to BRRE (CGTGYG) of the promoter to inhibit the transcription of BR synthesis genes. *BES1/BZR1* regulates thousands of target genes by binding to E-BOX or BRRE in plants [23,24,25,26,27].

Previous studies have shown that *BES1s* not only act as transcription factors for direct regulation, but also interact with other proteins to regulate target genes. *BES1* can inhibit the expression of *ABI3* and significantly downregulate the expression of downstream *ABI5*, inhibit ABA signal output, and promote seed germination [28,29,30]. BES1 interacts with G-protein β subunit AGB1 or auxin response element ARF6 and inhibits the expression of *GA2ox* to regulate cell elongation [31,32,33,34]. BES1 may bind to the promoter of ethylene synthesis gene *ACSs* to promote cell division in the root meristem and cell elongation in the mature zone [35]. UVR8 (UV RESISTANCE LOCUS 8) suppresses the DNA-binding activity of *BES1* by binding to BES1. The complex of UVR8 and BES1 accumulates in the nucleus and ultimately controls plant photomorphogenesis [36]. Deubiquitination of BES1 by UBP12/UBP13 promotes BR signaling and plant growth [37]. In addition, the *BES1* gene family participates in the response to biotic and abiotic stresses, for example, using the mutant bes1-D with a higher susceptibility to necrotic fungi to confirm that *BES1* is involved in pathogen defense against pathogens [38], promoting thermomorphogenesis by interacting with RD26 to inhibit its expression [39], and interacting with *WRKY46/54/70* or binding to the promoter of *PIF4* to inhibit drought response [40,41].

In addition to *Arabidopsis thaliana*, there have been numerous studies on the *BES1* gene family in other species. In maize, *ZmBES1/BZR1* positively regulates kernel size, and the seed size and weight increase significantly in plants overexpressed by *ZmBES1/BZR1-5* [42]. In rice, OsBZR1 protein interacts with 14-3-3 protein to affect BR signal transduction [43]. *OsBZR1* can also regulate plant structure by binding to the promoter of *OsMIR396d* and activating its transcription [44], and *OsBZR1* can directly bind to the BRRE motif located in the *AMT1* promoter region to modulate the ammonium transporters [45]. In soybean, *GmBEHL1* (*AtBES1/BZR1* homolog 1) regulates the number of nodules [46]. In tomato, *SlBZR1D* upregulates the expression of various stress-related genes and positively regulates salt tolerance [47]. In apple, there are 22 members of the *BES1* gene family [48], and *BES1* induces the expression of *MYB88* under pathogen attack. The overexpression of MdBES1 in plants leads to the downregulation of MdMYB88 expression and, consequently, reduces the plant resistance to pathogens [49].

To date, *BES1* gene family has been functionally explored and characterized in many plant species. However, little analysis has been performed on how these genes respond to stress conditions in *Cucurbita moschata*, as a member of the Cucurbitaceae, which has good economic benefits and nutritional value. Therefore, it is of great significance to study the *BES1s* in *C. moschata*.

## 2. Results

### 2.1. Identification of BES1 Gene Family Members in Cucurbitaceae

In this study, the following six Cucurbitaceae species were chosen for the analysis: cucumber (*Cucumis sativus*), melon (*Cucumis melo*), bottle gourd (*Lagenaria siceraria*), watermelon (*Citrullus lanatus*), silver-seed gourd (*Cucurbita argyrosperma*), and winter squash (*Cucurbita moschata*). TBtools software (v1.108) was used to compare and analyze the AtBES1 proteins with the BES1 proteins in above six Cucurbitaceae species. A total of 50 BES1 proteins were identified in Cucurbitaceae. Table S1 shows the individual names of the corresponding *BES1* genes, chromosome location, gene length, and coding sequence length. The length of proteins was distributed in two ranges, from 235 to 403 amino acids and from 668 to 800 amino acids, and their molecular weights (Mw) ranged from 24.57 to 45 kDa and from 72 to 89.74 kDa. The isoelectric point (pI) values of proteins ranged from 5.51 to 9.98. Notably, all identified BES1 proteins have GRAVY (Grand Average of Hydrophilicity) values less than 0, which means these proteins are hydrophilic. All BES1 proteins were predicted to be located in the nucleus.

### 2.2. Evolutionary Analysis of BES1 Gene Family in Cucurbitaceae

**Figure 1:**
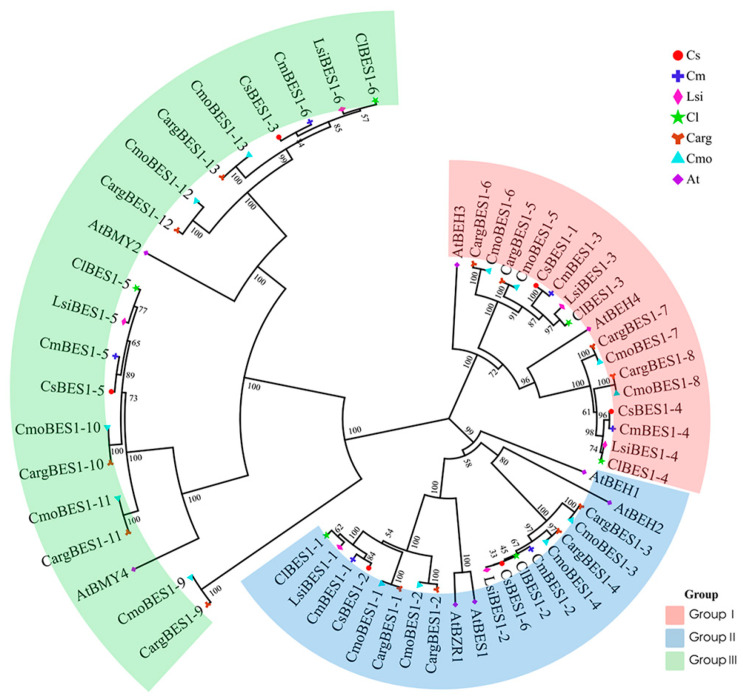
Phylogenetic relationships of BES1 proteins from cucumber (Cs), melon (Cm), bottle gourd (Lsi), watermelon (CI), silver-seed gourd (Carg), winter squash (Cmo), and *Arabidopsis thaliana* (At). All BES1 proteins in the seven species were clustered into three groups represented by pink, blue, and green for Groups I to III, respectively. The generated phylogenetic tree included 6 BES1 proteins from cucumber, 6 from melon, 6 from bottle gourd, 6 from watermelon, 13 from silver-seed gourd, 13 from winter squash, and 8 from *Arabidopsis thaliana*. Gene information can be found in Table S1.

To better explore the evolutionary relationship of BES1 proteins, a phylogenetic tree was constructed using MEGA11 (ClustalW, NJ methods) for a total of 58 BES1 members as study objects, of which 8 BES1 proteins belonged to *Arabidopsis thaliana* and 50 BES1 proteins in above six species belonged to Cucurbitaceae. As shown in Figure 1, these proteins were divided into three distinct groups based on their gene structures: Group I consisted of 18 BES1 members, Group II consisted of 20 BES1 members, and Group III consisted of 20 BES1 members. The close evolutionary relationship could be found between silver-seed gourd with winter squash, cucumber with melon, and bottle gourd with watermelon.

### 2.3. BES1s Members in Cucurbitaceae Showed Variations in Chromosomal Localization

**Figure 2:**
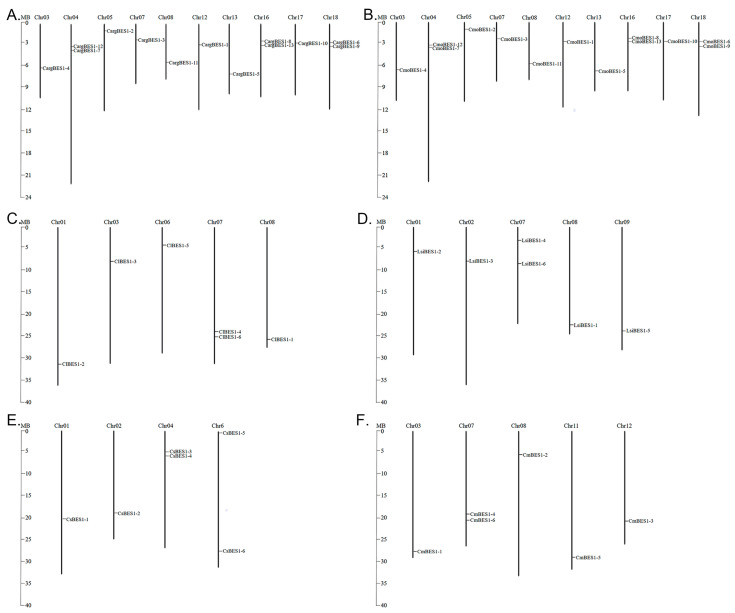
Localization of *BES1* genes on the chromosomes of six Cucurbitaceae species. (**A**) Localization of *CargBES1* genes on the chromosome of silver-seed gourd, (**B**) localization of *CmoBES1* genes on the chromosome of winter squash, (**C**) localization of *CIBES1* genes on the chromosome of watermelon, (**D**) localization of *LsiBES1* genes on the chromosome of bottle gourd, (**E**) localization of *CsBES1* genes on the chromosome of cucumber, and (**F**) localization of *CmBES1* genes on the chromosome of melon. The chromosome number was labelled on the top of each chromosome. The left scale represents the length of chromosomes, and scale is expressed in megabase (MB).

The localization of 58 *BES1* genes on the chromosomes in Cucurbitaceae was mapped using TBtools software. As shown in Figure 2, the *BES1s* of silver-seed gourd and winter squash were localized on 10 chromosomes, of which there were 2 *BES1s* on each of chromosomes 04, 16, and 18 (Figure 2A,B). The *BES1s* of watermelon, bottle gourd, and melon were localized on five chromosomes, of which there were two *BES1s* on chromosome 07 (Figure 2C,D,F). The *BES1s* of cucumber were localized on four chromosomes, of which there were two *BES1s* on chromosome 04 (Figure 2E). The localization of *BES1s* of silver-seed gourd and winter squash were almost identical on the same chromosome and had similar protein structures but differed significantly from other species. This suggests that the kinship distance between different Cucurbitaceae species has a significant influence on the localization of *BES1* genes.

As mentioned in a previous study [50], the entire chromosomes of *C. moschata* (allotetraploid, 2*n* = 40) were divided into two groups to represent two paleo-subgenomes, with 8 (chromosome 1, 2, 6, 7, 8, 11, 12, and 18) and 11 (chromosome 3, 5, 9, 10, 13, 14, 15, 16, 17, 19, and 20) chromosomes assigned to subgenomes A and B, respectively, and chromosome 4 was divided into three segments with two assigned to subgenome A and one to subgenome B. Combined with Figure 2B in this study, it could be speculated that seven (*CmoBES1-3, -11*, *-1*, *-6*, *-9*, *-7*, and *-12*) and six (*CmoBES1-4*, *-2*, *-5*, *-8*, *-13*, and *-10*) genes might be part of subgenomes A and B, respectively.

### 2.4. Analysis of Structures, Conserved Motifs, and Cis-Acting Elements

**Figure 3:**
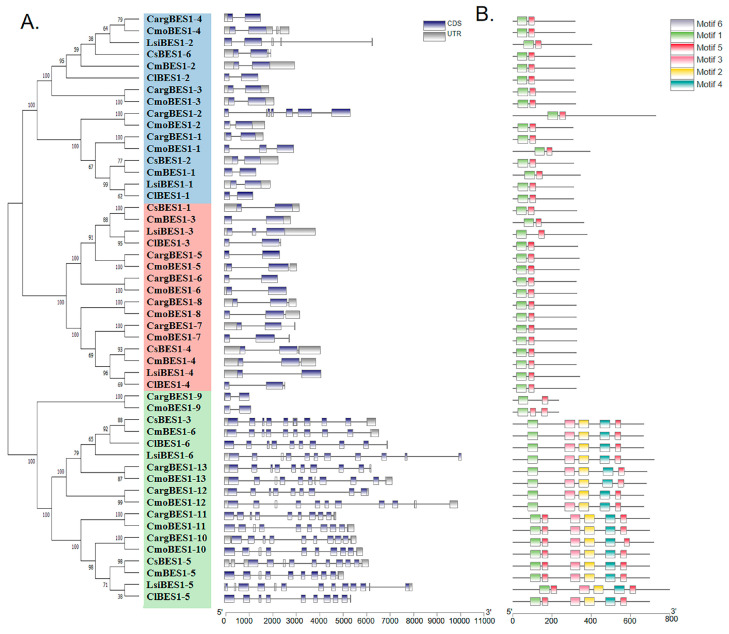
Gene structures (**A**) and conserved motifs distribution (**B**) of BES1s in six Cucurbitaceae species (silver-seed gourd, watermelon, cucumber, winter squash, bottle gourd, and melon). The CDS were represented by blue boxes, and the UTRs were represented by gray boxes. Motif analysis was conducted using the MEME online software (https://meme-suite.org/meme/tools/meme, accessed on 31 August 2022) and TBtools software (v1.108) as described in the Materials and Methods Section 4.4. Different color boxes represented various types of conserved motifs.

The structural characteristics of *BES1s*, consisting of CDS (coding DNA sequences), UTRs (untranslated regions), and introns, were mapped using TBtools software. As shown in Figure 3A, the family members contained at least 2 exons and up to 14 exons, with at least 1 intron and up to 13 introns. The conserved motifs of all BES1 amino acids in the above six species were analyzed using the MEME website. It was found that the number of motifs in Group III was significantly higher than that in Groups I and II (Figure 3B). Notably, all *BES1* families had motifs 1 and 5, in which Groups I and II had only these two motifs, while Group III had five or six motifs. These findings suggest that motifs 1 and 5 were highly conserved and might play the important role in the *BES1* gene family. Moreover, as shown in the phylogenetic tree, BES1 proteins exhibited similar motif composition with close evolutionary relationships.

**Figure 4:**
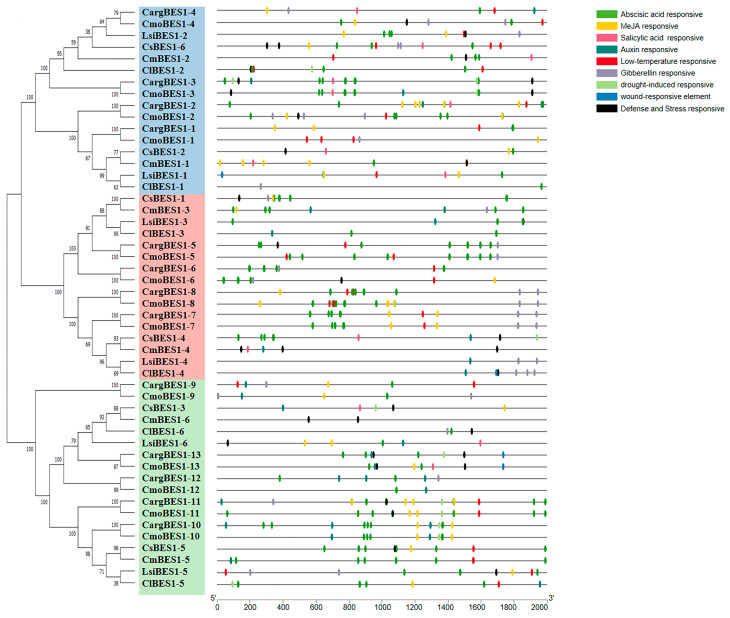
The distribution of stress-responsive *cis*-acting elements in the promoter regions of *BES1* genes of silver-seed gourd, watermelon, cucumber, winter squash, bottle gourd, and melon. The *cis*-acting regulators of the 2 Kbp sequence upstream of *BES1s* were predicted through the PlantCARE database website. The green, yellow, pink, dark green, red, gray, laurel green, blue, and black ovals represent abscisic acid response elements, MeJA response elements, salicylic acid response elements, auxin response elements, low-temperature response elements, gibberellin response elements, drought response elements, wound response elements, and defense and stress response elements, respectively.

The prediction of *cis*-acting elements may provide directions for studying the role of genes in plant growth and response to various biotic and abiotic stresses. We explored the *cis*-acting elements of CDS upstream 2 Kbp sequence of the *BES1* genes through the PlantCARE website and found that the *BES1* gene family contained many *cis*-acting elements that were responsive to abiotic stresses and phytohormones. In this study, nine relevant *cis*-acting elements were analyzed, including abscisic acid (ABA), MeJA, salicylic acid, auxin, gibberellin, low-temperature, drought, wound, and defense and stress-response elements. As shown in Figure 4, there were significant differences in the types and numbers of response elements among the six species, with the fewest in watermelon and most in silver-seed gourd and winter squash. A total of 263 ABA response elements were the most abundant, followed by 108 MeJA response elements and other *cis*-acting elements involved in stress and hormone responses. This result suggests that the *BES1* family might also participate in plant growth and stress responses.

### 2.5. Expression Analysis of CmoBES1 Family in C. moschata

**Figure 5:**
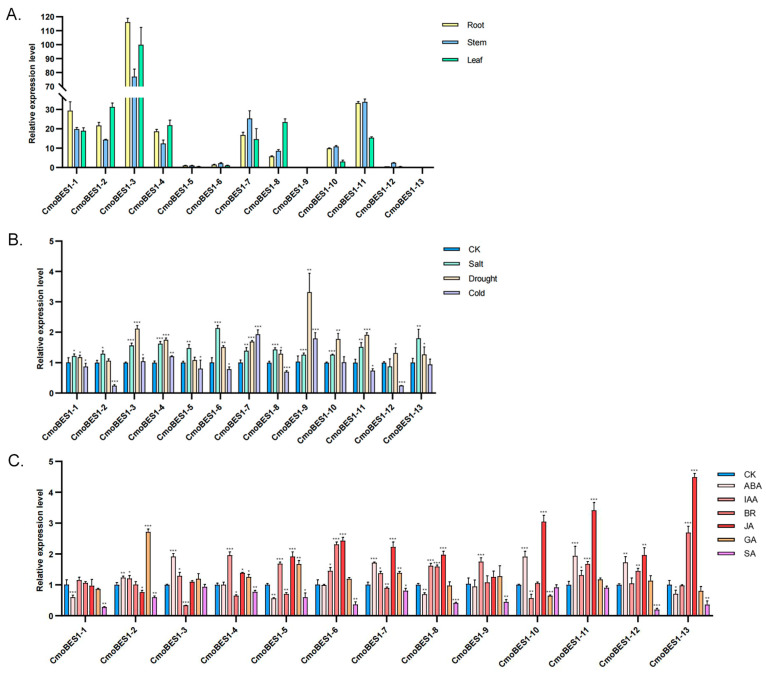
Expression patterns of 13 *CmoBES1* genes in different tissues and under treatments. (**A**) The expression pattern of 13 *CmoBES1* genes in roots, stems, and leaves. The expression of *CmoBES1-5* in leaves was set as 1. The expression of other *CmoBES1* genes were deduced with the expression of *CmoBES1-5* in leaves. (**B**) Relative expression levels of 13 *CmoBES1* genes under salt, drought, and cold treatments. (**C**) Relative expression levels of 13 *CmoBES1* genes under ABA, IAA, BR, JA, GA, and SA treatments. The average threshold cycle of qPCR as File S1 shown. The determined expression levels of all genes were calculated by the 2^−∆∆CT^ method. The expression of genes in CK (control group, Hoagland’s nutrient solution) was set as 1. The experimental information was described in the Materials and Methods Section 4.5. Error bars show the standard deviation of the three replicates, and the asterisk indicates a significant difference. (Student’s *t*-test; * *p* < 0.05; ** *p* < 0.01; *** *p* < 0.001.)

To reveal the function of the *CmoBES1* gene family in different tissues of *C. moschata*, the expression levels of *CmoBES1* transcripts in roots, stems, and leaves were inferred by quantitative real-time PCR (qRT-PCR). As shown in Figure 5A, *CmoBES1-3* had the highest levels in all tissues, whereas *CmoBES1-9* and *CmoBES1-13* had almost no levels in all tissues. Notably, three genes (*CmoBES1-2*, *-4*, and *-8*) were expressed at higher levels in leaves than in other tissues; five genes (*CmoBES1-6*, *-7*, *-10*, *-11*, and *-12*) showed relatively high expression levels in stems, which might promote stem elongation; and three genes (*CmoBES1-1*, *-3*, and *-11*) were relatively high in roots, which might promote root development.

The *BES1* gene family is involved in many stress-response processes; therefore, in this study, the expression levels of *CmoBES1* genes under three stresses (salt, drought, and cold) were investigated. As shown in Figure 5B, almost all the *CmoBES1* genes were significantly upregulated under salt and drought stress. Salt stress induced the expression of 12 *CmoBES1* genes at an extent from 1.21- to 2.13-fold, except for *CmoBES1-12*, which was not affected. Drought treatment significantly increased the transcript levels of 11 *ComBES1* genes except *CmoBES1-2* and *CmoBES1-5*. Cold stress induced the expression of *CmoBES1-4*, *-7*, and *-9* by 1.21-, 1.93-, and 1.79-fold, respectively, and reduced the expression of *CmoBES1-2*, *-5*, *-6*, *-8*, *-11*, and *-12* to 24.39%, 80.11%, 77.84%, 70.24%, 73.75%, and 24.88%, respectively. These results suggested that most of *CmoBES1s* positively responded to coordinate growth and defense.

The *BES1* gene family plays an important role in hormone pathways, so we examined the expression levels of *CmoBES1s* under six different hormones. As shown in Figure 5C, IAA induced the expression of nine genes (*CmoBES1-2*, *-3*, *-4*, *-5*, *-6*, *-7*, *-8*, *-9*, and *-11*) with distributions increased by 1.21-, 1.29-, 1.96-, 1.68-, 1.44-, 1.37-, 1.62-, 1.73-, and 1.31-fold, respectively, except *CmoBES1-10* reduced to 57%. ABA and BR regulated *CmoBES1* genes expression with no rules. ABA induced the expression of *CmoBES1-2*, *-3*, *-7*, *-10*, *-11*, and *-12* by 1.24-, 1.92-, 1.71-, 1.91-, 1.93-, and 1.73-fold, and reduced the expression of *CmoBES1-1*, *-5*, *-8*, and *-13* to 59.23%, 55.98%, 69.78%, and 70.75%, respectively. BR induced the expression of *CmoBES1-6*, *-8*, *-11*, *-12*, and -*13* by 2.28-, 1.59-, 1.66-, 1.44-, and 2.67-fold, and the expression levels of *CmoBES1-3*, *-4*, *-5*, and *-7* were inhibited by feedback to 33.44%, 64.75%, 70.24%, and 89.75%, respectively. There were differences in the regulation of *CmoBES1*s by JA, GA, and SA under JA treatment. The expression levels of nine genes (*CmoBES1-4*, *-5*, *-6*, *-7*, *-8*, *-10*, *-11*, *-12*, and *-13*) were upregulated by 1.39, 1.73-, 2.40-, 2.21-, 1.97-, 3.04-, 3.40-, 1.96-, and 4.46-fold, respectively, while *CmoBES1-2* was downregulated to 76%, and other three genes (*CmoBES1-1*, *-3*, and *-9*) showed no significant change. For GA treatment, the expression of *CmoBES1-2*, *-4*, *-5*, and *-7* were upregulated by 2.70-, 1.25-, 1.66-, and 1.38-fold, respectively, and the expression level of *CmoBES1-10* were inhibited by feedback to 64.65%. For SA treatment, the expression levels of 10 genes (*CmoBES1-1*, *-2*, *-4*, *-5*, *-6*, *-7*, *-8*, *-9*, *-12*, and *-13*) were inhibited by feedback to 27.84%, 59.71%, 77.40%, 59.33%, 36.27%, 80.55%, 41.57%, 43.13%, 19.99%, and 35.97%, respectively. All these results suggest that *BES1* gene family might regulate plant growth by responding to different hormones.

### 2.6. Subcellular Localization of ComBES1 Family

**Figure 6:**
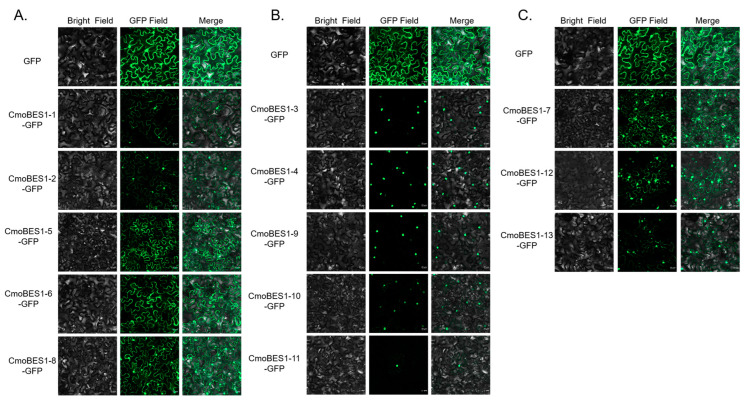
Subcellular localization of CmoBES1 proteins in *Nicotiana benthamiana* leaves. (**A**) CmoBES1-1, -2, -5, -6, -8-GFP proteins; (**B**) CmoBES1-3, -4, -9, -10, -11-GFP proteins; and (**C**) CmoBES1-7, -12, -13-GFP proteins. Each line contains a bright field, GFP field, and merged photos, and the empty GFP was the control. The length of the scale bar is 20 μm.

As shown in Figure 6, fluorescent signals of CmoBES1 proteins were observed in different subcellular locations of *Nicotiana benthamiana* epidermal cells.

Fluorescence signals of CmoBES1-1, -2, -5, -6, and -8 were detected in the nucleus and cytoplasm, suggesting they might have the function as transcription factors (Figure 6A). Fluorescence signals of CmoBES1-3, -4, -9, -10, and -11 were detected in the nucleus, and CmoBES1-10 and CmoBES1-11 also had faint signal in the cytoplasm (Figure 6B). CmoBES1-7, -12, and -13 proteins were mainly localized to the nucleus and cytoplasm, as well as possibly to some complex organelles, such as vesicle-like structural organelles and chloroplasts (Figure 6C). We further validated the results in maize protoplasts using the same transformation method (Figure S1), with the difference that the localization of CmoBES1-7, -12, and -13 on other organelles was not obvious.

**Figure 7:**
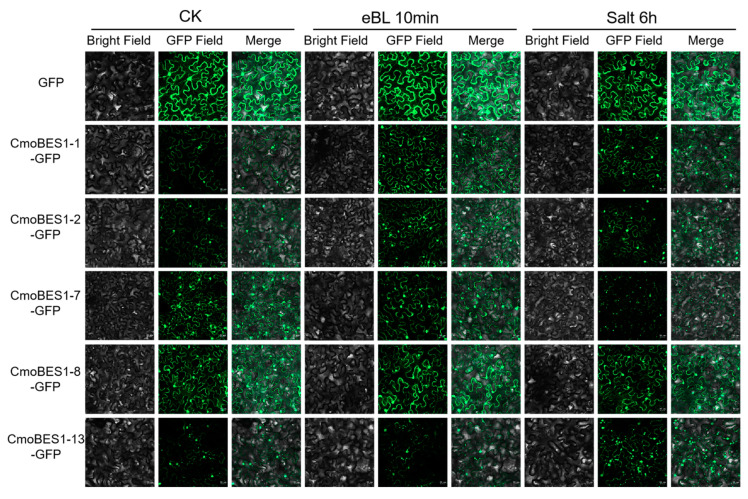
Subcellular localization of CmoBES1 proteins under eBL and salt treatments in *Nicotiana benthamiana* leaves. Each line contains the bright field, GFP field, and merged photos of CmoBES1-1, -2, -7, -8-GFP, and GFP control. The length of the scale bar is 20 μm.

After eBL and salt treatments, the fluorescence signals of CmoBES1-1, -2, -7, and -8 changed (Figure 7). The signals of CmoBES1-1 and CmoBES1-2 increased, while the signals of CmoBES1-7 and CmoBES1-8 increased only under BR treatment. Interestingly, under salt treatment, the fluorescence of CmoBES1-7 in the nucleus and cytoplasm was reduced, while the fluorescence of CmoBES1-13 increased. It was difficult to judge whether the fluorescence of other CmoBES1 proteins changed (Figure S2). Therefore, it could be seen that some CmoBES1 proteins responded to the BR signaling pathway and some responded to salt stress, suggesting that different BES1 proteins might play unique roles involving different or identical signaling pathways.

### 2.7. Transactivation Assay

**Figure 8:**
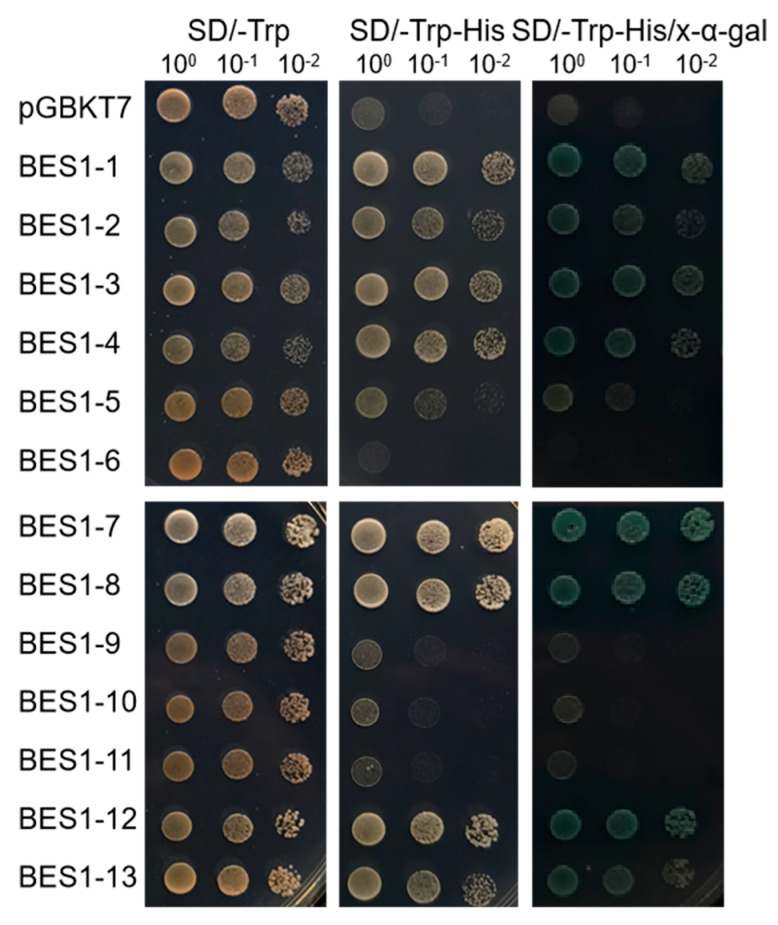
Transactivation assays of 13 BES1 proteins in yeast cells. The transformed yeast grown on SD/-Trp media or SD/-Trp-His media. LacZ activity was assessed by β−galactosidase filter lift assay. Empty vector pGBKT7 was used as a negative control. The length of the scale bar is 4 mm.

To identify the transcriptionally active proteins in the CmoBES1 proteins, the transcriptional activity experiment was performed using yeast cell that harboring pGBKT7 vector as a negative control. As shown in Figure 8, CmoBES1-1, -2, -3, -4, -7, -8, -12, and -13 yeast cells grew well on the SD/-Trp-His medium and showed β-galactosidase activities. CmoBES1-5 yeast cells grew at a low rate as they had lower transcriptional activity, while CmoBES1-6, -9, -10, and -11 yeast cells could not grow properly on the SD/-Trp-His medium, which means they had no transcriptional activity in yeast cells. This might be due to the presence of transcriptional repression domains in the full-length transcription factor.

## 3. Discussion

Transcription factors can control chromatin and transcription by recognizing specific nucleotide sequences and guide genome expression in complex systems, playing vital roles in plant growth and resistance [1]. The *BES1* transcription factors family activate or inhibit thousands of genes through their specific sequences, integrating multiple signals to regulate plant development and environmental adaptations [7]. In our study, 13, 13, 6, 6, 6, and 6 BES1 protein sequences were identified in silver-seed gourd, winter squash, cucumber, melon, bottle gourd, and watermelon, respectively (Table S1). The Mw of all BES1 proteins in Cucurbitaceae showed two ranges: from 24.57 to 45 kDa and from 72 to 89.74 kDa. It is worth noting that all identified BES1 proteins may play a conserved role as hydrophilic proteins. These proteins were divided into three group according to gene structures and as reported in *Arabidopsis thaliana* [51]. Notably, all BES1 family members had motifs 1 and 5, Groups I and II had only these 2 motifs, Group III had more motifs, and closely related BES1 members in the phylogenetic tree had common motifs. These findings suggested that motif 1 and motif 5 were highly conserved and might play an important role in the BES1 gene family. Moreover, the functions of BES1 proteins in the same group are similar. We also identified and analyzed 13 ComBES1 proteins’ characteristics that shared the same conserved domains and were quite similar to other species [4]. The location and number of motifs in the same branch were similar. Silver-seed gourd had a close evolutionary relationship with *C. moschata*, which was consistent with the analysis of the phylogenetic tree.

Promoters contain important *cis*-acting elements for gene initiation and transcription regulation [52]. The *BES1* gene family is essential in many stress responses [10,39,40,41]. The prediction of *cis*-acting elements can provide directions for investigating the response effects of *BES1* genes to various biotic and abiotic stresses. In this study, many *cis*-acting elements of *BES1s* were predicted in Cucurbitaceae (Figure 3), with the largest number of ABA-responsive elements, followed by MeJA-responsive elements, indicating that the expression of *BES1s* was mainly regulated by ABA and JA, and other different *cis*-acting elements were also involved. The expression levels of *BES1s* in *C. moschata* under four abiotic stresses and six hormones were investigated. Most *CmoBES1* genes were significantly regulated under salt, drought, and cold stresses (Figure 5), indicating that *CmoBES1* genes family exhibited expression variations in response stress treatments. Under the treatments of IAA, ABA, JA, GA, and SA, the expression of 9, 6, 9, 4, and 0 genes in the *CmoBES1 gene* family were significantly up regulated, respectively, and 1, 4, 1, 1, and 10 genes were significantly feedback inhibited, respectively. Within these genes, different *CmoBES1* genes differently responded to stresses, which might be related to the *cis*-acting elements contained in the upstream promoter. Combined with the prediction of *cis*-acting elements, we analyzed the expression of the *CmoBES1* gene family under stresses and found that *CmoBES1-12* was not induced by salt treatment, *CmoBES1-2* and *CmoBES1-5* were not induced by drought treatment, and *CmoBES1-10* and -*13* did not change significantly under cold treatment, which was consistent with the absence of corresponding *cis*-acting elements on their gene promoters. Under the treatment of ABA, IAA, JA, and SA, three different *BES1* genes were no significant, and no or rarely cis-acting elements were found on the corresponding promoter. The case of GA treatment was similar, in which the promoter region of *CmoBES1* genes without significant changes did not contain or only fewer GA response sites. In addition, a large number of previous studies have demonstrated that BES1 transcription factors regulate root development, cell division, plant architecture, and plant photomorphogenesis [31,35,53]. Our research also showed that *CmoBES1* exhibited different expression patterns in different tissues (Figure 5A); notably, some *CmoBES1* genes showed specific expression levels in roots, stems, and leaves, illustrating that different *BES1* genes played specific functions in promoting root development, stem elongation, and leaf growth and indicating that plant growth requires different genes to interact and coordinate. These results indicate that the *CmoBES1* family may regulate plant growth by responding to different hormones.

Furthermore, the subcellular localization of the CmoBES1 proteins showed that 10 proteins were localized in the cytoplasm and nucleus (Figure 6), which was different from the predicted protein localization (Table S1). We thought that the reason might be that the protein sequences was regulated by a variety of factors to exert their functions at specific locations in vivo. BRs play important roles in plant growth processes [4,6,35,37]. The *BES1* gene family can interact with other proteins or bind directly to nucleic acids to regulate target genes and plays an important role in the BR signaling pathway [15,27]. In our study, we demonstrated that *CmoBES1* transcriptional expression was regulated by BRs, which induced the upregulation of five *CmoBES1* genes and feedback inhibition of four *CmoBES1* genes’ expression levels (Figure 6C). Moreover, we found that eight CmoBES1 proteins were mainly located in the nucleus and cytoplasm (Figure 6) and had strong transcriptional activity (Figure 8), suggesting that they might function as transcription factors. Finally, combined with subcellular localization experiments, four CmoBES1 proteins were found to respond to BR (Figure 7), indicating that these four *CmoBES1* might be the major transcription factors involved in the BR signaling pathway in *CmoBES1* gene family, and might be downstream components of the BR signaling pathway. Our analysis of BES1s diversity, localization, and expression in Curcubitaceae contributes to the better understanding of the essential roles of these transcription factors in plants.

## 4. Materials and Methods

### 4.1. Identification of BES1s Gene Family Members in Cucurbitaceae

We downloaded the protein sequence database of cucumber (*Cucumis sativus*, diploid, 2*n* = 14), melon (*Cucumis melo*, diploid, 2*n* = 24), bottle gourd (*Lagenaria siceraria*, diploid, 2*n* = 22), watermelon (*Citrullus lanatus*, diploid, 2*n* = 22), silver-seed gourd (*Cucurbita argyrosperma*, diploid, 2*n* = 20), and winter squash (*Cucurbita moschata*, allotetraploid, 2*n* = 40) from the Cucurbitaceae genome database (http://cucurbitgenomics.org/, accessed on 31 August 2022). In addition, we downloaded the protein sequences of all AtBES1s from the phytozome database (https://phytozome-next.jgi.doe.gov/, accessed on 31 August 2022) and compared the AtBES1 proteins with the proteins of the above six species using the program ClustalW in software MEGA7 (v11.0.10) to obtain candidate BES1s protein sequences. The domains of candidate BES1 proteins were obtained by the Pfam domain database (http://pfam-legacy.xfam.org/search#tabview=tab1, accessed on 31 August 2022) and the Conserved domains database (https://www.ncbi.nlm.nih.gov/cdd/?term=, accessed on 31 August 2022). All BES1 protein sequences were identified by removing sequences, excluding the BES1-N domain.

### 4.2. Physicochemical Properties and Chromosomal Localization Analysis

The length and CDS sequence of the *BES1* genes and the location of this gene on chromosome were available from the Cucurbitaceae website and visualized using TBtools software. The ExPASy website (https://web.expasy.org/protparam/, accessed on 31 August 2022) was used to predict the physicochemical properties of BES1 proteins, including the relative molecular mass (Mw), isoelectric point (pI), and amino acid, etc. The subcellular localization of BES1 proteins was predicted using the BUSCA website (http://busca.biocomp.unibo.it/, accessed on 31 August 2022) and the WoLF PSORT website (https://wolfpsort.hgc.jp/, accessed on 31 August 2022).

### 4.3. Evolutionary Analysis

Using the Program ClustalW in the software MEGA7 (v11.0.10), all protein sequences of Cucurbitaceae BES1 were compared with *AtBES1* proteins, and the phylogenetic tree of the Cucurbitaceae BES1 family was constructed using the neighbor-joining (NJ) method and the self-help method of phylogenetic experiments (Bootstrap method, Bootstrap = 1000, and the *p*-distance model). The tree was visualized and optimized through the ChiPlot (https://www.chiplot.online/#Phylogenetic-Tree, accessed on 1 September 2022).

### 4.4. Gene Structure, Conserved Motifs and Cis-Acting Regulating Element Prediction

To investigate the genetic structure of *BES1*, gff3 files of six species were downloaded from the Cucurbitaceae database, and the conserved motifs of BES1 amino acids were analyzed and identified using the MEME website (https://meme-suite.org/meme/tools/meme, accessed on 1 September 2022), with the number of conserved domains set to 6. *Cis*-acting regulator upstream of the 2 Kbp sequence upstream of Cucurbitaceae *BES1s* was used to predict the PlantCARE database website (https://bioinformatics.psb.ugent.be/webtools/plantcare/html/, accessed on 1 September 2022). The above results were visualized using TBtools software (v1.108).

### 4.5. Plant Materials, Abiotic Stress Treatment and Expression Data

In this experiment, the “TianMiyihao” was used as the experimental material, seedlings were planted in plastic tubes with Hoagland’s nutrient solution in a greenhouse (28 °C, 16 h light/8 h dark, 70–80% humidity), and the outside of all tubes was wrapped with tin foil. When the plants were in the three-leaf stage, we collected *C. moschata* roots, stems, and leaves to analyze the tissue expression patterns. Each sample was taken from three different plants, and three biological replicates were performed. Meanwhile, the three-leaf plants were treated with the following conditions: 20% PEG6000 (20 g PEG6000, use the Hoagland’s nutrient solution up to 100 mL), 150 mM NaCl (0.876 g NaCl, use the Hoagland’s nutrient solution up to 100 mL), 4 °C, and 100 μM different hormones (ABA, IAA, JA, GA, SA, or eBL, 1 M, add into Hoagland’s nutrient solution), CK (control group, distilled water add into Hoagland’s nutrient solution). Leaves of each group were harvested after 6 h of treatment, frozen in liquid nitrogen and stored at −80 °C. Each sample was taken from three different plants and three biological replicates were performed.

Total RNA was extracted from the sample using Trizol reagent (Takara, Beijing, China), and the first strand of cDNA was obtained by the reverse transcribing of 1 µg of RNA according to the manufacturer’s First-Strand cDNA Synthesis Kit (Vazyme, Nanjing, China). Expression levels were evaluated by qRT-PCR, and the primers for the *ComBES1* gene were designed using Primer Premier 5 software (v5.00) (Table S2). The reaction mix contained 10 µL AceQ qPCR SYBR Green Master Mix (Vazyme, Nanjing, China), 0.4 µL upstream primers, 0.4 µL downstream primers, 2 µL cDNA, and up to 20 µL with ddH_2_O. The qRT-PCR process was set as follows: stage 1 was the initial denaturation for 30 s at 95 °C; stage 2 was circular reaction at 95 °C for 10 s and 60 °C for 30 s, 40 cycles; stage 3 was melting curve at 95 °C for 15 s, 60 °C for 1 min. The average threshold cycle (Ct) for each sample was calculated, the determined transcript abundance of genes was calculated by the 2^−∆∆CT^ method [54], and β-actin was used as an internal control. Three biological replicates and three experimental replicates were performed for each sample.

### 4.6. Gene Clone, Recombinant Plasmid Construction, Subcellular Localization, and Transcriptional Activity Analysis

The *CmoBES1* genes were amplified from *C. moschata* three-leaf stage leaves using their specific primers (Table S3), and the cloned fragments were connected to Blunt-Zero Vector (Vazyme, Nanjing, China) and sequenced. For subcellular localization and transcriptional activity analysis, the *CmoBES1* gene-coding sequence without terminating codons was amplified using the primers (Tables S4 and S5), and the correct sequence was introduced into pCAMBIA1305 and pGBKT7 vector with GFP tags by homologous recombination to form the recombinant plasmids.

For transient expression, *N. benthamiana* was used as an experimental host; it has good efficiency in gene transformation and regeneration and has been demonstrated to be effective in transient expression of a variety of proteins [55,56,57,58]. The 2 mL of resuspended *Agrobacterium* strain GV3101 carrying *CmoBES1*-GFP and GFP vector were injected into 3–4-week-old *N. benthamiana* leaves. Previous studies have implicated that BES1 proteins regulated the expression of target genes by altering their phosphorylation status, thereby shuttling between the cytoplasm and nucleus and participating in the BR signaling pathway [25,49]. Therefore, in this experiment, after dark infiltration for 2 d, *N. benthamiana* was sprayed with 10 μM eBL for 10 min and poured into 150 mM saline for 6 h, and the green fluorescence of BES1 protein was conserved at 488 nm using a confocal microscope (Zeiss, Jena, Germany).

For transcriptional activity analysis, pGBKT7 vector and 13 *ComBES1*-pGBKT7 were transformed into yeast strain Y1HGold cells, respectively, and their transcriptional activities were determined by observing their growth status on SD/-Trp, SD/-Trp-His, and SD/-Trp-His with X-α-Gal medium, as described in Zhu’s report [59].

## 5. Conclusions

In this study, *BES1s* were identified in six Cucurbitaceae species and functionally characterized in *C. moschata*. The 13 *CmoBES1* genes exhibited different expression patterns in three tissues, suggesting that different *BES1* genes perform specific functions in promoting root development, stem elongation, and leaf growth. The combination of *cis*-elements and *CmoBES1* family experiments under stress and hormone treatment suggests that the *CmoBES1* gene family might regulate plant growth and development by responding to different hormones. Most of CmoBES1 proteins were localized in the nucleus and cytoplasm. Combining the transcriptional activity with the subcellular localization change, four CmoBES1 proteins were found to respond to BR, indicating that these four *CmoBES1* genes might be the major transcription factors of the downstream components of BRs signaling in the *CmoBES1* gene family. Our study provides a basis for further studies on the role of *CmoBES1s* in Cucurbitaceae.

## Data Availability

All data are displayed in the manuscript and Supplementary Files.

## Supplementary Materials

The following supporting information can be downloaded at: https://www.mdpi.com/article/10.3390/ijms24032287/s1.
