# Supplementary material for: Genome-Wide Identification of BES1 Gene Family in Six Cucurbitaceae Species and Its Expression Analysis in Cucurbita moschata
Source: Int J Mol Sci. 2023 Jan 24;24(3):2287. doi: 10.3390/ijms24032287 (PMC9916444; doi:10.3390/ijms24032287)
Supplement: Supplementary file 1 [file ijms-24-02287-s001.zip › supplementary Figtures.pdf]

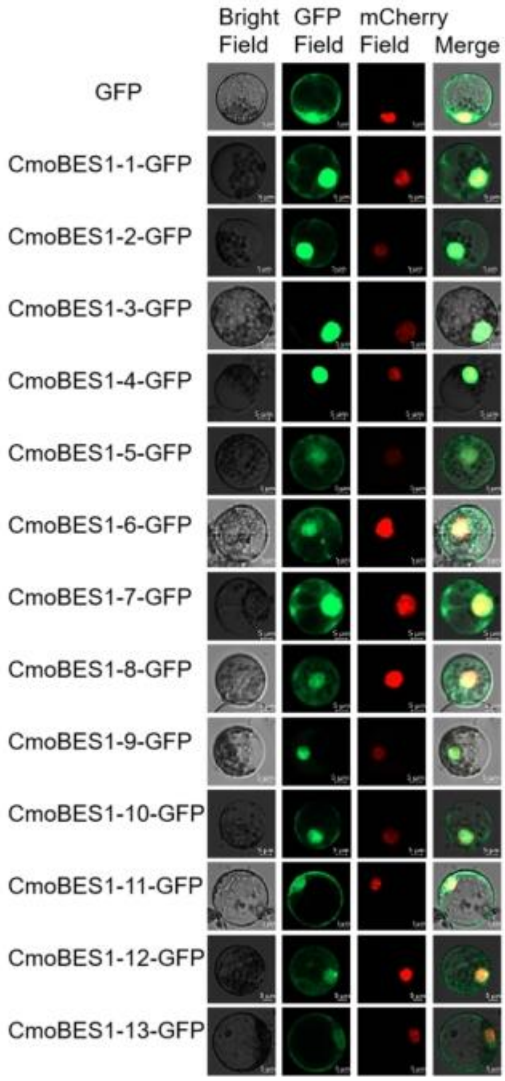

Figure S1. Subcellular localization of CmoBES1 proteins in maize protoplasts. Each line contains Bright field, GFP field, the nuclei in which the mCherry signal field and Merged photos of *CmoBES1*-GFP or GFP control. The length of Scale bar is 5 μm.

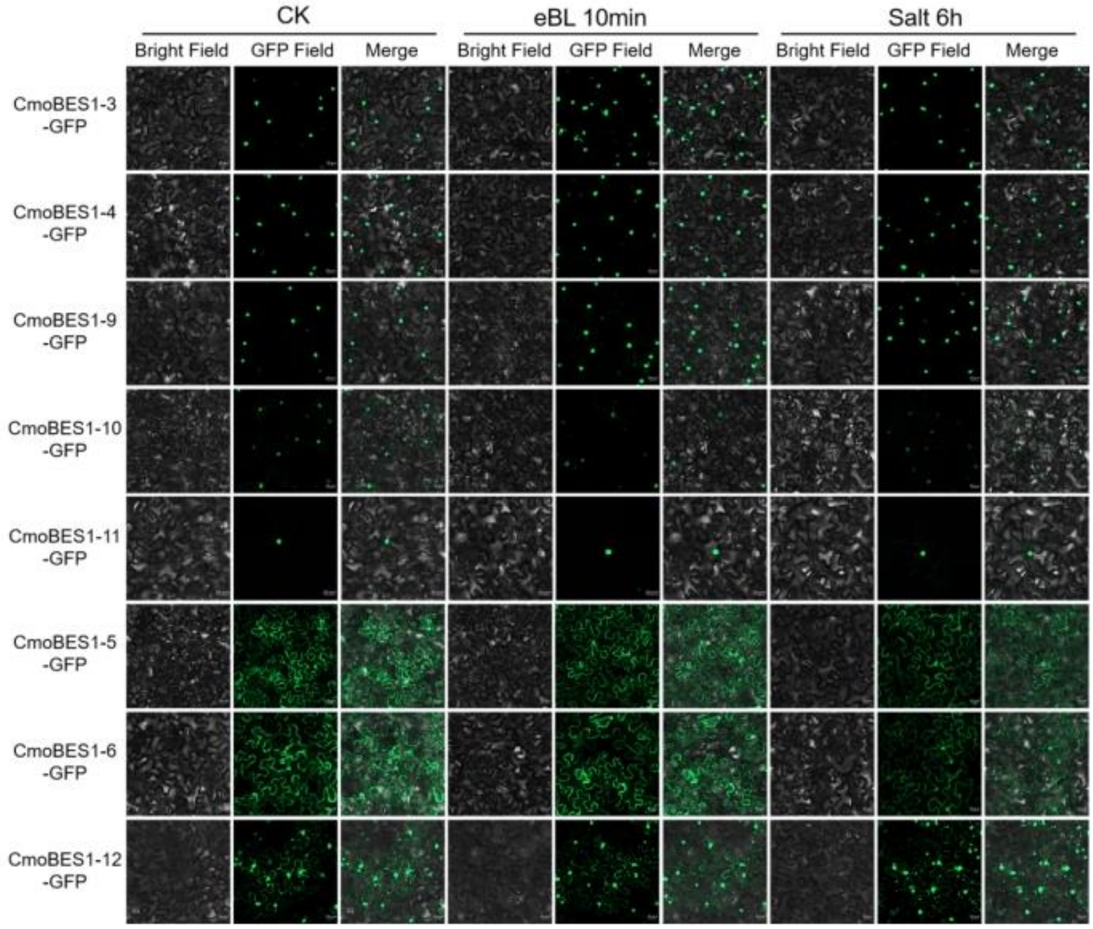

Figure S2. Subcellular localization of CmoBES1 proteins under eBL and Salt treatments in Nicotiana benthamiana leaves. Each line contains Bright field, GFP field, and Merged photos of *CmoBES1*-GFP and GFP control. The length of Scale bar is 20 μm.
