# Supplementary material for: Genome-Wide Identification of BES1 Gene Family in Six Cucurbitaceae Species and Its Expression Analysis in Cucurbita moschata
Source: Int J Mol Sci. 2023 Jan 24;24(3):2287. doi: 10.3390/ijms24032287 (PMC9916444; doi:10.3390/ijms24032287)
Supplement: Supplementary file 1 [file ijms-24-02287-s001.zip › supplementary Tables.pdf]

Table S1. Identification of *BES1* gene family members in Cucurbitaceae.

Species	Gene name	Gene ID	PI	MW (kDa)	Chromosome Location	Gene Length (bp)	CDS Length (bp)	Number of Amino Acids (aa)	GRAVY	Subcellular Localization
Silver-seed gourd	*CargBES1-1*	Carg04068-RA	8.95	34.05901	Chr07 : 1680987 .. 1682802 (-)	1816	933	310	-0.685	nucleus
	CargBES1-2	Carg11815-RA	9.47	80.77915	Chr03 : 256033 .. 261904 (-)	5872	2193	730	-0.353	nucleus
	CargBES1-3	Carg16031-RA	8.94	35.22832	Chr12: 289584 .. 291652 (+)	2069	972	323	-0.711	nucleus
	CargBES1-4	Carg00664-RA	8.96	34.68366	Chr05 : 869666 .. 871362 (-)	1697	960	319	-0.666	nucleus
	CargBES1-5	Carg19900-RA	9.03	72.04139	Chr13 : 171866 .. 174460 (+)	2595	1029	342	-0.58	nucleus
	CargBES1-6	Carg14565-RA	8.76	35.17207	Chr16 : 333270 .. 335766 (-)	2497	981	326	-0.662	nucleus
	CargBES1-7	Carg13090-RA	9.04	35.32433	Chr04 : 242329 .. 245630 (+)	3302	990	329	-0.576	nucleus
	CargBES1-8	Carg11361-RA	8.74	34.74362	Chr18 : 484338 .. 487696 (-)	3359	978	325	-0.567	nucleus
	CargBES1-9	Carg22574-RA	9.02	24.78738	Chr04 : 451819 .. 452979 (-)	1161	714	237	-0.622	nucleus
	CargBES1-10	Carg05599-RA	5.63	80.33348	Chr08 : 1560855 .. 1566992 (-)	6138	2157	718	-0.383	nucleus
	CargBES1-11	Carg27097-RA	5.7	78.31012	Chr16 : 101816 .. 107025 (+)	5210	2100	699	-0.454	nucleus
	CargBES1-12	Carg13149-RA	5.95	75.60825	Chr17 : 611288 .. 618006 (-)	6719	2013	670	-0.392	nucleus
	CargBES1-13	Carg11425-RA	5.89	76.93148	Chr18 : 971696 .. 978533 (-)	6838	2058	685	-0.401	nucleus
Winter squash	*CmoBES1-1*	CmoCh12G004350.1	9.98	43.95144	Chr12 : 2676074 .. 2678979 (-)	2906	1188	395	-0.682	nucleus
	CmoBES1-2	CmoCh05G002340.1	8.64	33.73464	Chr05 : 1002966 .. 1004658 (+)	1693	930	309	-0.67	nucleus
	CmoBES1-3	CmoCh07G005060.1	9.05	35.25642	Chr07 : 2298841 .. 2300921 (-)	2081	972	323	-0.713	nucleus
	CmoBES1-4	CmoCh03G008060.1	8.96	34.6556	Chr03 : 6530547 .. 6533260 (-)	2714	960	319	-0.674	nucleus
	CmoBES1-5	CmoCh13G006090.1	8.95	36.72911	Chr13 : 6674594 .. 6677626 (-)	3033	1029	342	-0.585	nucleus
	CmoBES1-6	CmoCh18G004120.1	8.89	35.42932	Chr18 : 2705280 .. 2707890 (+)	2611	987	328	-0.671	nucleus
	CmoBES1-7	CmoCh04G007120.1	8.89	35.24322	Chr04 : 3530555 .. 3533287 (-)	2733	990	329	-0.569	nucleus
	CmoBES1-8	CmoCh16G004630.1	8.74	34.74362	Chr16 : 2222424 .. 2225580 (-)	3157	978	325	-0.567	nucleus
	CmoBES1-9	CmoCh18G004840.1	9.15	24.56718	Chr18 : 3331106 .. 3332209 (-)	1104	708	235	-0.589	nucleus
	CmoBES1-10	CmoCh17G004070.1	5.51	78.08286	Chr17 : 2702840 .. 2708637 (-)	5798	2097	698	-0.386	nucleus
	CmoBES1-11	CmoCh08G008810.1	5.59	78.03185	Chr08 : 5705362 .. 5710787 (+)	5426	2097	698	-0.419	nucleus
	CmoBES1-12	CmoCh04G006380.1	5.78	75.62812	Chr04 : 3153817 .. 3163603 (+)	9787	2013	670	-0.418	nucleus
	CmoBES1-13	CmoCh16G005570.1	5.92	76.69929	Chr16 : 2713632 .. 2720668 (-)	7037	2052	683	-0.394	nucleus
Cucumber	*CsBES1-1*	CsaV3_1G033010.1	8.97	35.34841	Chr01 : 20054584 .. 20057911 (-)	3328	984	327	-0.644	nucleus
	CsBES1-2	CsaV3_2G028510.1	9.17	34.18614	Chr02 : 18745171 .. 18747558 (+)	2388	936	311	-0.715	nucleus
	CsBES1-3	CsaV3_4G007080.1	5.91	75.25573	Chr04 : 4795128 .. 4801821 (+)	6694	2010	669	-0.404	nucleus
	CsBES1-4	CsaV3_4G008150.1	8.5	34.68454	Chr04 : 5720049 .. 5724301 (+)	4253	978	325	-0.572	nucleus
	CsBES1-5	CsaV3_6G000500.1	5.78	78.34708	Chr06 : 340089 .. 346465 (-)	6377	2097	698	-0.441	nucleus
	CsBES1-6	CsaV3_6G045980.1	8.96	34.63861	Chr06 : 27211404 .. 27213475 (+)	2072	960	319	-0.689	nucleus

Species	Gene name	Gene ID	PI	MW (kDa)	Chromosome Location	Gene Length (bp)	CDS Length (bp)	Number of Amino Acids (aa)	GRAVY	Subcellular Localization
Melon	*CmBES1-1*	MELO3C010925.1	9.26	37.73998	Chr03 : 29999092 .. 30000475 (-)	1384	1038	345	-0.677	nucleus
	CmBES1-2	MELO3C007804.1	8.96	34.71269	Chr08 : 5468519 .. 5471484 (+)	2966	960	319	-0.697	nucleus
	CmBES1-3	MELO3C002681.1	9	39.5201	Chr12 : 22058946 .. 22061841 (-)	2896	1098	365	-0.604	nucleus
	CmBES1-4	MELO3C016121.1	8.5	34.70054	Chr07 : 20822745 .. 20826732 (-)	3988	978	325	-0.583	nucleus
	CmBES1-5	MELO3C021214.1	5.74	78.20803	Chr11 : 31280641 .. 31285843 (-)	5203	2097	698	-0.429	nucleus
	CmBES1-6	MELO3C016213.1	5.9	75.11352	Chr07 : 22268773 .. 22275513 (+)	6741	2007	668	-0.398	nucleus
Bottle gourd	*LsiBES1-1*	Lsi08G014140.1	9.17	34.04504	Chr08 : 22379317 .. 22381295 (-)	1979	936	311	-0.674	nucleus
	LsiBES1-2	Lsi01G007040.1	8.9	44.48891	Chr01 : 5623250 .. 5629639 (-)	6390	1212	403	-0.704	nucleus
	LsiBES1-3	Lsi02G008360.1	9.11	41.16632	Chr02 : 7840220 .. 7844147 (+)	3928	1143	380	-0.443	nucleus
	LsiBES1-4	Lsi07G002850.1	8.65	36.92698	Chr07 : 3072923 .. 3077087 (+)	4165	1035	344	-0.582	nucleus
	LsiBES1-5	Lsi09G015620.1	6.01	89.73852	Chr09 : 23742072 .. 23750179 (-)	8108	2403	800	-0.340	nucleus
	LsiBES1-6	Lsi07G007700.1	8.07	81.78941	Chr07 : 8391546 .. 8401766 (+)	10221	2172	723	-0.414	nucleus
Watermelon	*ClBES1-1*	Cla97C08G158860.1	9.12	34.012	Chr08 : 26100260 .. 26101552 (-)	1293	936	311	-0.669	nucleus
	ClBES1-2	Cla97C01G018680.1	8.66	34.11202	Chr01 : 31863038 .. 31864549 (+)	1512	942	313	-0.677	nucleus
	ClBES1-3	Cla97C03G058680.1	8.97	35.71687	Chr03 : 7987903 .. 7990456 (-)	2554	1002	333	-0.608	nucleus
	ClBES1-4	Cla97C07G136850.1	8.71	34.72858	Chr07 : 24325969 .. 24328700 (-)	2732	978	325	-0.611	nucleus
	ClBES1-5	Cla97C06G113190.1	5.66	78.23406	Chr06 : 4199370 .. 4205071 (+)	5702	2097	698	-0.423	nucleus
	ClBES1-6	Cla97C07G137900.1	5.94	75.32282	Chr07 : 25559689 .. 25567044 (-)	7356	2010	669	-0.396	nucleus

Table S2. qRT-PCR primers for expression analysis of *CmoBES1* genes.

Primer name	Sequence (5'to3')
CmoBES1-1-qF	TCGTTACCCCCACCCCTCAC
CmoBES1-1-qR	CTCTCCTCCGCCGCTCTCTG
CmoBES1-2-qF	AACGACGAAGGAGAGCCATC
CmoBES1-2-qR	ACCAGCATCAGCACAGAGGG
CmoBES1-3-qF	CTTCCACTACACCTCCGTTC
CmoBES1-3-qR	ATTGATTCCTCTTCCACCAC
CmoBES1-4-qF	TAACCTCTTTCCGCCATCCA
CmoBES1-4-qR	AAGCGTCCGACTCGTCACAT
CmoBES1-5-qF	GCTGGGTGGACTGTAGAGGA
CmoBES1-5-qR	AGACGCAGGACTTGGGTTAT
CmoBES1-6-qF	CGACTTCCCACCATTTTTTC
CmoBES1-6-qR	CGCCGCTTGTTATTCTCCCT
CmoBES1-7-qF	CTTGTTTCTACTAACCCATT
CmoBES1-7-qR	TTCCTGCGGCATTGCTTCCA
CmoBES1-8-qF	GCTTCCCAAACATTGCGACA
CmoBES1-8-qR	CCACCAACCACATCCATACG
CmoBES1-9-qF	AGGGAAAGGCAAAGAAGAGC
CmoBES1-9-qR	TAGATGCGGAGGAGCGATAG
CmoBES1-10-qF	GCTCAGTCCACTTCTCTTAG
CmoBES1-10-qR	TGACAATAAATATGGACTCG
CmoBES1-11-qF	CTTGGAAAGACATAACCTCG
CmoBES1-11-qR	AACACCATTTACAACTGCTG
CmoBES1-12-qF	AGACGATTTGGTGCCCTGTT
CmoBES1-12-qR	CTTCTGGTTGTGGTTGTTGC
CmoBES1-13-qF	GAAGTAGGACTGGGGGCATC
CmoBES1-13-qR	GCACGCCATTTGTTGACCAT
CmoBES1-β-Actin-qF	GTGCCTGCTATGTATGTTGCC
CmoBES1-β-Actin-qR	GGTCCAAACGGAGAATGGCATG

Table S3. The primers for cloning *CmoBES1* genes.

Primer name	sequence (5'to3')
CmoBES1-1-F	TCGTTACCCCCACCCCTCAC
CmoBES1-1-R	TCAAGCCTTACTGCTACTACC
CmoBES1-2-F	ATGACCTCCGACGGCGCCACTTC
CmoBES1-2-R	CTTGTCGGGCTCTGCCATAA
CmoBES1-3-F	CTCTGTCTCTTCGCCGCTCG
CmoBES1-3-R	ATTGATTCCTCTTCCACCAC
CmoBES1-4-F	AAGTTTGCTTGGTGATGCGT
CmoBES1-4-R	GCGAACCAGGGTCTTTCTAATG
CmoBES1-5-F	GCACCGTTTTACAGTGATTTTG
CmoBES1-5-R	GCCAACCAATACACACGGACAT
CmoBES1-6-F	CGACTTCCCACCATTTTTTC
CmoBES1-6-R	TTAAGAGGATGTAACTGGAT
CmoBES1-7-F	ATGACGTCGGGGACGAGGCTAC
CmoBES1-7-R	TTATAACTTAAATCTGGTCCT
CmoBES1-8-F	ATGACGTCGGGGACGAGGCTAC
CmoBES1-8-R	GGATTTGCTGAGTAACTATTTG
CmoBES1-9-F	ATGAAGGAAGGAAGTGAAGTTGG
CmoBES1-9-R	CTACGAACGAAGCCGTGGAGATC
CmoBES1-10-F	ATGGCAACGGATATGCAAAAATTG
CmoBES1-10-R	TGACAATAAATATGGACTCG
CmoBES1-11-F	ATGGCAACAAATATGCAAAAAATG
CmoBES1-11-R	AACACCATTTACAACTGCTG
CmoBES1-12-F	ATGAGCGGCAGCTTGAACGAC
CmoBES1-12-R	GAGAGGAGAACAAAACACCC
CmoBES1-13-F	CAGAGGAGAGCTGCAATCGGAA
CmoBES1-13-R	CTTCAACTGGGCACCAAATCGCC

Table S4. The primers for construction of *CmoBES1*-GFP vectors.

Primer name	sequence (5'to3')
CmoBES1-1-GFP-F	ggacagcccagatcaactagtCTCGTTACCCCCACCCCTC
CmoBES1-1-GFP-R	ggtcctcgagacgtctctagaAGCCTTACTGCTACTACCAAGTGTGA
CmoBES1-2-GFP-F	ggacagcccagatcaactagtATGACCTCCGACGGCGCC
CmoBES1-2-GFP-R	ggtcctcgagacgtctctagaATCAAAACAAGGTTTCTTACTGTTG
CmoBES1-3-GFP-F	ggacagcccagatcaactagtATGCCTGAAGAAGCCATGACA
CmoBES1-3-GFP-R	ggtcctcgagacgtctctagaACCACGGGCCTTCCCACT
CmoBES1-4-GFP-F	ggacagcccagatcaactagtATGACGGGCAGAGGGTCATC
CmoBES1-4-GFP-R	ggtcctcgagacgtctctagaACAACGGGCCTTCCCACC
CmoBES1-5-GFP-F	ggacagcccagatcaactagtGCACCGTTTTACAGTGATTTTGTT
CmoBES1-5-GFP-R	ggtcctcgagacgtctctagaCCTCGTCCTTGAATTCCCAAG
CmoBES1-6-GFP-F	GACAGCCCAGATCAACTAGTCGACTTCCCACCATTTTTTC
CmoBES1-6-GFP-R	ggtcctcgagacgtctctagaAGAGGATGTAACTGGATTAAC
CmoBES1-7-GFP-F	ggacagcccagatcaactagtATGACGTCGGGGACGAGG
CmoBES1-7-GFP-R	ggtcctcgagacgtctctagaTCCTGCGGCATTGCTTCC
CmoBES1-8-GFP-F	ggacagcccagatcaactagtATGACGTCGGGGACGAGG
CmoBES1-8-GFP-R	ggtcctcgagacgtctctagaTCTGGTTCTTGAGTTCCCAAGAG
CmoBES1-9-GFP-F	ggacagcccagatcaactagtATGAAGGAAGGAAGTGAAGTTGGT
CmoBES1-9-GFP-R	ggtcctcgagacgtctctagaCGAACGAAGCCGTGGAGA
CmoBES1-10-GFP-F	ggacagcccagatcaactagtATGGCAACGGATATGCAAAAA
CmoBES1-10-GFP-R	ggtcctcgagacgtctctagaTACCTGGAGATCATGAACAGCTTC
CmoBES1-11-GFP-F	ggacagcccagatcaactagtATGGCAACAAATATGCAAAAAATG
CmoBES1-11-GFP-R	ggtcctcgagacgtctctagaTACCTGGAGATCATGAACAGATTCTC
CmoBES1-12-GFP-F	ggacagcccagatcaactagtATGAGCGGCAGCTTGAACG
ComBES1-12-GFP-R	ggtcctcgagacgtctctagaACAGGGCACCAAATCGTCTACT
CmoBES1-13-GFP-F	ggacagcccagatcaactagtATGAGCGGCAGCTTAAACGA
CmoBES1-13-GFP-R	ggtcctcgagacgtctctagaACTGGGCACCAAATCGCC

Table S5. The primers for construction of *CmoBES1*- pGBKT7 vectors.

Primer name	sequence (5'to3')
CmoBES1-1-pGBKT7 -F	atggccatggaggccgaattcCTCGTTACCCCCACCCCTC
CmoBES1-1-pGBKT7 -R	ctagttatgcggccgctgcagAGCCTTACTGCTACTACCAAGTGTGA
CmoBES1-2-pGBKT7 -F	atggccatggaggccgaattcATGACCTCCGACGGCGCC
CmoBES1-2-pGBKT7 -R	ctagttatgcggccgctgcagATCAAAACAAGGTTTCTTACTGTTGTTA
CmoBES1-3-pGBKT7 -F	atggccatggaggccgaattcATGCCTGAAGAAGCCATGACA
CmoBES1-3-pGBKT7 -R	ctagttatgcggccgctgcagACCACGGGCCTTCCCACT
CmoBES1-4-pGBKT7 -F	atggccatggaggccgaattcATGACGGGCAGAGGGTCATC
CmoBES1-4-pGBKT7 -R	ctagttatgcggccgctgcagACAACGGGCCTTCCCACC
CmoBES1-5-pGBKT7 -F	atggccatggaggccgaattcGCACCGTTTTACAGTGATTTTGTT
CmoBES1-5-pGBKT7 -R	ctagttatgcggccgctgcagCCTCGTCCTTGAATTCCCAAG
CmoBES1-6-pGBKT7 -F	atggccatggaggccgaattcCGACTTCCCACCATTTTTTCA
CmoBES1-6-pGBKT7 -R	ctagttatgcggccgctgcagAGAGGATGTAACTGGATTAACCCTTG
CmoBES1-7-pGBKT7 -F	atggccatggaggccgaattcATGACGTCGGGGACGAGG
CmoBES1-7-pGBKT7 -R	ctagttatgcggccgctgcagAATTCCTGCGGCATTGCTT
CmoBES1-8-pGBKT7 -F	atggccatggaggccgaattcATGACGTCGGGGACGAGG
CmoBES1-8-pGBKT7 -R	ctagttatgcggccgctgcagTCTGGTTCTTGAGTTCCCAAGAG
CmoBES1-9-pGBKT7 -F	atggccatggaggccgaattcATGAAGGAAGGAAGTGAAGTTGGT
CmoBES1-9-pGBKT7 -R	ctagttatgcggccgctgcagCGAACGAAGCCGTGGAGA
CmoBES1-10-pGBKT7 -F	atggccatggaggccgaattcATGGCAACGGATATGCAAAAA
CmoBES1-10-pGBKT7 -R	ctagttatgcggccgctgcagTACCTGGAGATCATGAACAGCTTC
CmoBES1-11-pGBKT7 -F	atggccatggaggccgaattcATGGCAACAAATATGCAAAAAATG
CmoBES1-11-pGBKT7 -R	ctagttatgcggccgctgcagTACCTGGAGATCATGAACAGATTCTC
CmoBES1-12-pGBKT7 -F	atggccatggaggccgaattcATGAGCGGCAGCTTGAACG
CmoBES1-12-pGBKT7 -R	ctagttatgcggccgctgcagACAGGGCACCAAATCGTCTACT
CmoBES1-13-pGBKT7 -F	atggccatggaggccgaattcATGAGCGGCAGCTTAAACGA
CmoBES1-13-pGBKT7 -R	ctagttatgcggccgctgcagACTGGGCACCAAATCGCC
